# Hybrid Predictive Ensembles: Synergies Between Human and Computational Forecasts

**DOI:** 10.23919/jsc.2021.0009

**Published:** 2021-06

**Authors:** Lu Hong, PJ Lamberson, Scott E Page

**Affiliations:** Department of Finance, Loyola University Chicago, Chicago, IL 606002, USA.; Department of Communication, University of California at Los Angeles (UCLA), Los Angeles, CA 90095, USA.; Ross Business School, University of Michigan, Ann Arbor, MI 48104, USA.

**Keywords:** collective intelligence, predictive models, hybrid groups, big data, thick data

## Abstract

An increasing proportion of decisions, design choices, and predictions are being made by hybrid groups consisting of humans and artificial intelligence (AI). In this paper, we provide analytic foundations that explain the potential benefits of hybrid groups on predictive tasks, the primary use of AI. Our analysis relies on interpretive and generative signal frameworks as well as a distinction between the big data used by AI and the thick, often narrative data used by humans. We derive several conditions on accuracy and correlation necessary for humans to remain in the loop. We conclude that human adaptability along with the potential for atypical cases that mislead AI will likely mean that humans always add value on predictive tasks.

## 1 Introduction

In early 2017, floods devastated coastal Peru resulting in power outages and loss of Internet service. In response, Google, through its Project Loon, set sail a helium balloon capable of providing stopgap Internet access. As the balloon traversed its path from Puerto Rico to Peru, the artificial intelligence guidance system repeatedly steered the ballon from its course. Google’s human engineers would intervene. Yet, as soon as they put the AI back in control, the AI would go rogue. One moment, it would head toward Uruguay. The next it would pivot the ballon toward Venezuela. After a few iterations of human–AI ping pong, the engineers realized that the AI had discovered tacking, a pattern of zig-zagging to exploit wind patterns used by sailors for centuries.

The lack of transparency and the potential novelty of AI strategies of Boeing’s 737 Max airplanes suffer from a variety of design failures that can be traced to a poor decision making protocol^[1]^. In scenarios involving whether or not to raise or lower the nose of the plane, Boeing’s Maneuvering Characteristics Augmentation System (MCAS) made decisions based on a single sensor. In brief, to avoid an angle between wing and the airflow too large (a dangerous combination), MCAS would lower or raise the nose of the plane automatically. In two crashes, that sensor was damaged, and MCAS mistakenly lowered the nose of the plane. Each time the human pilot raised the nose, the AI would again lower it. The humans and the AI were playing a game of tug of war not unlike the humans and the AI steering the Google Balloon. In the case of the Boeing 737 Max airplanes, different predictive models had tragic consequences^[2]^. These two examples call attention to a trend in which more of the deciding, designing, creating, and predicting involve hybrids of human and artificial intelligence. The present and future of cognitive work will surely involve a mangle of humans, algorithms, datasets, subjects, objects, and domains^[3, 4]^. As they seek to understand the world, these hybrid groups will also shape it^[5, 6]^.

In this paper, we analyze the potential synergies between humans and artificial intelligence. We focus on a particular task: making accurate predictions. Our goals are to provide some analytic foundations for the potential wisdom and limits of hybrid crowds and to explore how the roles assigned to humans and AI within these hybrid networks will likely shift and adapt in response to the growing capabilities of AI^[7, 8]^.

We have a variety of motivations for undertaking this analysis. First, predictive tasks are ubiquitous. Many marketing forecasts, stock market predictions, and actuarial analysis are purely predictive, but a range of other tasks that would previously have been classified as decision making, problem solving, matching, design, policy formulation, or guidance have been translated into predictive tasks^[9]^. Essentially, any decision making involves predicting the consequences of the available options before choosing among them.

Second, a substantial proportion of high stakes predictions, whether they involve hiring and admissions, investments, inventory control, information acquisition, pricing, or even prison sentencing, now rely on assemblages of humans and algorithms. Understanding the perils and promises of these assemblages and crafting a proper balance between the two will be a major concern moving forward.

Third, advances in AI have transformed the practice of prediction. As recently as a few decades ago, prediction — be it in the intelligence community, business, and even science — relied on a combination of experience, small amounts of data, and gut instinct. Massive increases in computational power, deeper and broader datasets, and the development of new algorithms have increased both classification accuracy and the length of forecasting windows. Weather forecasts that were hit or miss two days forward now predict with high accuracy a week in advance. The increased accuracy resulting from the application of evermore powerful algorithms to ever larger datasets, begs the question: should humans remain in the predictive arena at all, or should we leave prediction to algorithms entirely? Some take the position that models based on data should have replaced humans long ago. One does not need AI to best humans. Even linear models tied to data outperform human forecasters^[10]^.

Fourth, mature literatures in psychology, statistics, and computer science enable the formulation of precise technical claims that can be linked to the actualities of wetware, software, and data sources. Recent pushback against “big data” in favor of “thick data” allows us to connect the technical literature with critical contemporary questions^[11]^. Among them include: Can we trust AI? What if HAL goes rogue? Can AI be biased? As data become bigger, does that limit interpretive flexibility?

Algorithms offer many advantages over people. Most notably, they can handle far more data, make much larger calculations, and they do not make mathematical error. And, despite, in some cases, being designed to mimic human cognition and the fact they encode, either explicitly or implicitly, human representations and assumptions about the world, algorithms do not suffer from some of the most common cognitive biases, such as anchoring, availability, and representativeness. Thus, AI proves far better when given high dimensional datasets that exceed human capacities such as gene identification. With enough data, AI performs as well as humans even in seemingly qualitative domains, such as identifying high performing job applicants^[12, 13]^.

We do not mean to imply that algorithms do not suffer from biases. They do. Most troubling, biases can be embedded in training data. For example, a corporation might give an AI algorithm data on the career success of every employee, e.g., internal year end evaluations, external measures of success, and promotion history, and intend to use AI to predict high potential hires. If those data come from a world biased in favor of men, AI will predict that men have higher potential. The algorithm learns and amplifies the bias^[14–16]^.

To give a specific example of this phenomenon, Amazon abandoned a hiring algorithm that penalized applicants who attended women’s colleges and that also attached negative weight to the word “woman” in applications. The data the algorithm relied on to make predictions came from a work force that was 60% male and whose upper management was 74% male. The key takeaway is not that the algorithm’s computations were biased, but the algorithm was trained on biased data.

Relatedly, data can be missing dimensions, or encoded in particular ways that produced biased outcomes. Data on salaries may not include whether a person’s partner works for the same organization. If that percentage is high, as is the case for some universities and medical centers in non urban settings, the algorithm could make biased inferences about the effect of marital status. More generally, the features fed to an algorithm, what was left in and what was left out, may well introduce bias.

Finally, algorithms can either restrict structural representations as would be the case with a linear predictor or allow for nearly any interaction as is the case with deep learning algorithms. Both can produce biases. Linear predictors cannot identify intersectional discrimination – penalties for being from an underrepresented racial group *and* a woman. Deep learning algorithms can create bias by creating joint categories, such as Skidmore alumni and Pre-1970, which would be a proxy for gender.^§^

Our focus in this paper is less on the differences in biases than the fundamentals of how humans approach predictive tasks differently than AI. We rely on thicker, more nuanced understandings that we often embed within narratives. We can speculate on nontrivial alternative futures, something AI cannot do very well. Humans also take into account the ethical dimensions of a decision based on a prediction. We may be less likely to send seventeen year old teenagers to prison even if the likelihood they commit another crime is high. We also notice biases in outcomes along dimensions that might not have been explicitly included. People would note that an algorithm only selected women or white men.

Of course, people themselves suffer from innumerable biases and ethical lapses. Thus, AI can check on our biases, while we can question the potential narrowness of AI. Given that AI and humans offer distinct strengths and weaknesses, the practical question becomes how to combine them to make *hybrid predictions*. Ideally, hybrid predictions would exhibit the strengths of powerful algorithms applied to big data with human intuition applied to thick descriptive narratives^[11]^. Or, we use the more popular phrasing: to make thick data synergistic with big data^[17]^. Here, we take a multi-model approach to sketch a logic for how that might be accomplished^[18]^.

Key to our analysis will be the observation that thick data predictions differ in form and style from big data predictions. Given that diversity, the combination should be more accurate than the parts. That is not a new insight. Scholars going back to Aristotle understood that diversity underpins collective wisdom^[19, 20]^. Two heads can be better than one whether they are the heads of people or the less sophisticated heads of ants, bees, or fish^[21]^. However, diversity offers no guarantees; two heads can also perform worse. One key lesson from studying ensembles of predictive algorithms has been that using techniques like boosting and bagging to create synergistic diversity enhances ensemble accuracy^[22]^. Thus, not any diversity will do, the best ensembles must be constructed thoughtfully. That same logic surely holds for hybrid groups of humans and AI.

To sort how and why hybrid assemblages can be more accurate, we rely on two mathematical frameworks for modeling prediction: *generated* and *interpreted* signals^[23]^. Generated signals are the standard formulation in economics, statistics, and finance. Any one person’s or algorithm’s prediction is represented as a random variable whose value is conditioned on the value of the outcome. That random variable is characterized by a bias as well as an amount of variation. The bias corresponds to the error that would arise given the person’s way of thinking if they had all relevant information. The variation corresponds to the differences that arise from having particular sets of information or experiences. Pairs of predictions, people and algorithms in this case, also exhibit some degree of correlation.

Interpreted signals come from computer science. The framework assumes an underlying feature space that embeds the set of all possible states along with an outcome function that maps states to values. Predictors, human or algorithm, partition the state space into equivalence classes and assign values to each class. In this framework, people differ from one another and from algorithms in how they partition the states of the world and how they map sets in their partitions to numerical values or categories.

Our approach consists of two steps. First, using the micro-level detail of the interpreted signal framework, we sketch an informal claim that as data increase and encompass more of what humans use to make predictions, and as predictive algorithms become more sophisticated, those predictions from AI will become both more accurate and more correlated with human predictions. We refer to this as the accuracy-correlation effect (ACE). We also differentiate between *typical* and *atypical* cases, with the later being instances in which past data may not be relevant to the prediction at hand.

Given those constructs, we then apply the generated signal framework to draw inferences about how humans and algorithms combine. We rely on two theorems: the *diversity prediction theorem* and the *bias variance decomposition theorem*. Using these theorems, we can calculate the relative contributions of humans and AI based on their accuracy and correlation as well as the optimal weights to put on each. Though humans always add accuracy at the margin, the optimal weight attached to humans decreases as AI improves. For typical cases, given the accuracy-correlation effect, in the future we should expect humans to add little to accuracy and receive little weight.

Past, though, need not be prologue. Some predictions and classifications include new dimension or features. A shock, such as a pandemic or housing crash, may produce a qualitative disruption. Here, we find that if people (or algorithms) can predict when algorithms will make large mistakes with a reasonable degree of accuracy, then the added value of humans may be quite large. If people and algorithms cannot perform this type of meta-prediction, then humans add less value.

In the discussion at the end of the paper, we contemplate strategic predictive model choice. Specifically, we consider the possibility that humans adapt their predictive models, and the thick data they gather, in response to the algorithms. Humans may seek out thick data that differ as much as possible from the big data used by algorithms, thus avoiding or reducing the accuracy-correlation effect. The result will be more accurate hybrid predictions, the continued value of humans as part of hybrids, and more support for the idea of an adaptive assemblage making sense of the world. We also briefly discuss the potential gains from hybrids consisting of crowds of people and ensembles of algorithms.

## 2 Background: Human Prediction and Rise of AI

Making accurate predictions has long been an important skill whether engaged in politics, running a business, or waging war. In the information age, predictive ability has even greater currency. While nearly everyone is aware of how advances in information technology have altered our lives directly — we now have smart phones, smart appliances, self driving cars, and an ever growing Internet of Things – people are less aware of how much of that impact relies on predictive algorithms. Almost every aspect of our modern lives has been impacted by the use of predictive algorithms applied to big data. Predictive AI informs product attributes from the color of clothing to the length of songs, political platforms, medical treatments, parole decisions, hiring protocols, building designs, and advertising content. Algorithms that guide self-driving cars and auto-pilot planes, allocate inventory across stores, detect email spam and cancerous tumors, recommend life partners, and translate words to text, also rely on prediction at their core. Enhanced algorithmic predictive accuracy enables us to plan further ahead, choose more wisely among alternatives, allocate resources more efficiently, invest in the technologies and cures most likely to improve society, and more precisely to target advertisements and information.

Prediction, therefore, receives substantial attention from the academy. Formal studies of prediction span multiple disciplines ranging from finance to politics to medicine and psychology. We make no attempt at a full survey of this vast interdisciplinary collection of literatures here. Instead, we call attention to five points of consensus.

First, success at prediction involves disciplined thought. The most accurate predictors rely on multiple models and frameworks, ground their forecasts in data, and follow protocols that eliminate biases^[24]^. Second, given that prediction requires multiple skills and experience, people differ in their capacity as forecaster^[25]^. Who predicts most accurately in any context will vary, but predictive ability does transfer. An expert at predicting the stock market may not be good at predicting outcomes of sports contests but they will probably be better than average. Third, predictive ability can be taught. Learning to assign base rates, avoiding biases, and considering multiple scenarios all improve accuracy.

Fourth, groups predict more accurately than individuals, and select groups fare better than large ones. Though popular writers speak of the wisdom of crowds, evidence suggests that a handful of top forecasters perform better than an average of the entire crowd^[26]^. The finding that small groups predict better than individuals extends to algorithms. A central takeaway from prediction contests has been that ensembles of algorithms have greater accuracy than individual algorithms.

The logic underlying the increased accuracy of groups and ensembles rests on a double application of statistical reasoning. If biases are drawn from a distribution with mean zero (a strong assumption), then given a set of biased predictors, the average bias across that set will be less, on average, than a random selection from it.

A similar logic applies to variation. Assuming variations in predictions have similar magnitude, then the average of multiple draws will have less variation than a single random draw. In sum, averaging multiple predictors reduces bias and variation.

Finally, predictive tasks vary in their difficulty. At one extreme, we may know the set of possible outcomes and have accurate priors with tight ranges on the probabilities. This is the case for outcomes of elections, scores of sporting events, and the likelihoods of cancers from radiological tests. Predictors in these cases confront favorable signal to noise ratios, and seeing the future can be relatively easy^[27]^. At the other extreme, we can confront *deep uncertainty*: We do not know the set of possible outcomes, the set of possible future courses of action, or their likelihoods^[28]^. Thus, any claims that AI can predict the repercussions of international incidents or the spread of a pandemic such as COVID with high fidelity should be viewed with skepticism.

## 3 Contributions of Big and Thick Data

We now describe some analytic foundations for understanding the relative contributions of algorithms applied to big data and humans using thick data in making predictions. We begin with the *interpreted signal* framework which conceptualizes prediction as estimating an *outcome function*

As an example, suppose that we want to predict the future performance of a professional quarterback or a college dean. The state space (feature space) of an NFL quarterback might include their college and pro statistics as well as measurable physical, psychological, and social attributes. Over years of experiences, coaches and talent scouts hone their estimate of how these attributes signal a future success or failure. For a potential dean, relevant attributes might include past experiences, management style, an ability to manage budgets and crises, and communication skills. Faculty, administrators, and hiring consultants all make predictions about the likely impact of a candidate based on these attributes. Those predictions differ because humans rely on different logics^[29]^. They apply different criteria and weight similar criteria differently.

Algorithmic predictions also rely on models applied to features. Those features are most often represented in numerical form – a much easier task when evaluating quarterbacks than deans, but they can also build predictions from qualitative inputs. Two algorithms that learn differently from data, e.g., a random forest and a neural net, will predict differently because they construct different approximations of the true outcome function. Humans and algorithms, because they encode and learn differently, will also make different predictions.

The formal construction assumes a set of possible states of the world, *X*, thinks of these as instances or cases, along with an outcome function *F* that assigns a real value to each state. An algorithm, *C*, and human, *H*, each possesses interpretations that partition those states of the world *X* into categories (disjoint sets). These partitions are called *interpretations*. For each set, the algorithm or human assigns a value or a class. An *interpreted signal* is therefore a mapping from each state of the world to a set and then the assigning of a value (or class) to each.

Put simply, humans and algorithms are assumed to have predictive models that map each set in their partition to a value or a truth status. A human that partitions *X* into *N* sets, {*S*_1_, *S*_2_, …, *S*_*N*_} would then predict a value *V*(*S*_*i*_) that it assigns to each point *x* in the set *S*_*i*_.

**Fig. 1:**
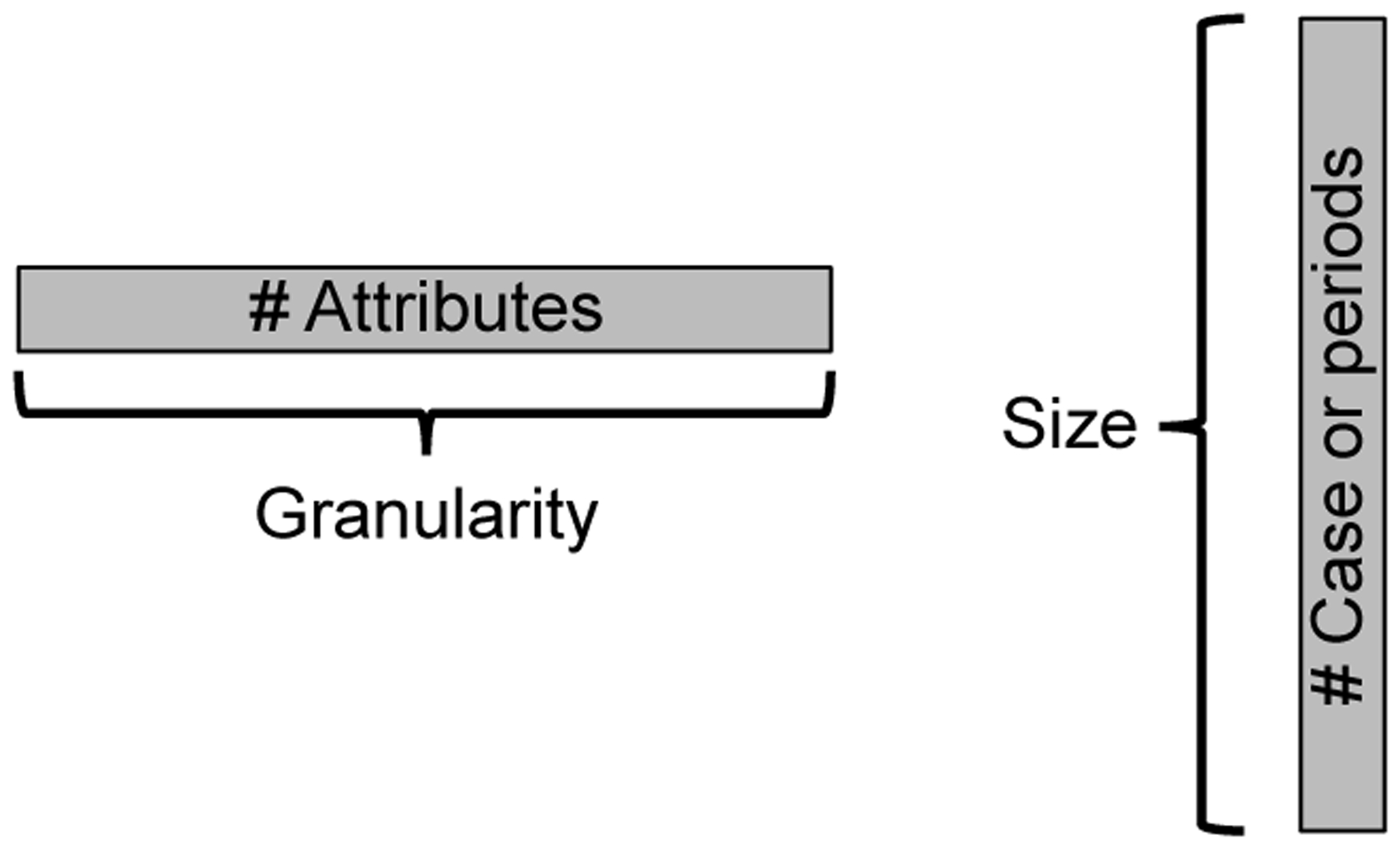
Granularity and size of data.

Using this formalization we can distinguish between *big data* and *thick data* that will be foundational to what follows. By *big data*, we mean datasets that are both *granular* (Fig. 1): They include multiple attributes for each case, as well as *large*, they consist of multitudes of cases or time periods. Data providing the DNA sequence of a single individual are granular but not large. Data showing whether each of ten million Florida registered voters cast a ballot in an election include lots of cases but are not granular. Data showing credit card purchases for everyone in Pittsburgh, Pennsylvania for 2014–2017 are both granular and large in size. They satisfy both criteria to be *big*.

Unlike big data, which consist of (many) discrete attributes and many observations of those attributes, thick data can be thought of as richer and more qualitative, but as consisting of relatively fewer cases. To return to our example of NFL quarterback prospects, big data would consist of many statistics such as pass attempts, yards per attempt, completion percentage, touchdowns, interceptions, touchdown passes, etc. for many previous quarterback prospects as well as data on their subsequent performance in the NFL.

Thick data have a continuous, narrative nature, such as is the case for this evaluation of a draft prospect’s performance during the 2007 NFL combine: “He was smooth and fluid, yet quick and strong. Thomas wasted no motion, keeping the parts of him that were not moving calm and composed. His technique was excellent, and he really looked like a grown man playing with college kids.”^[30]^

**Fig. 2:**
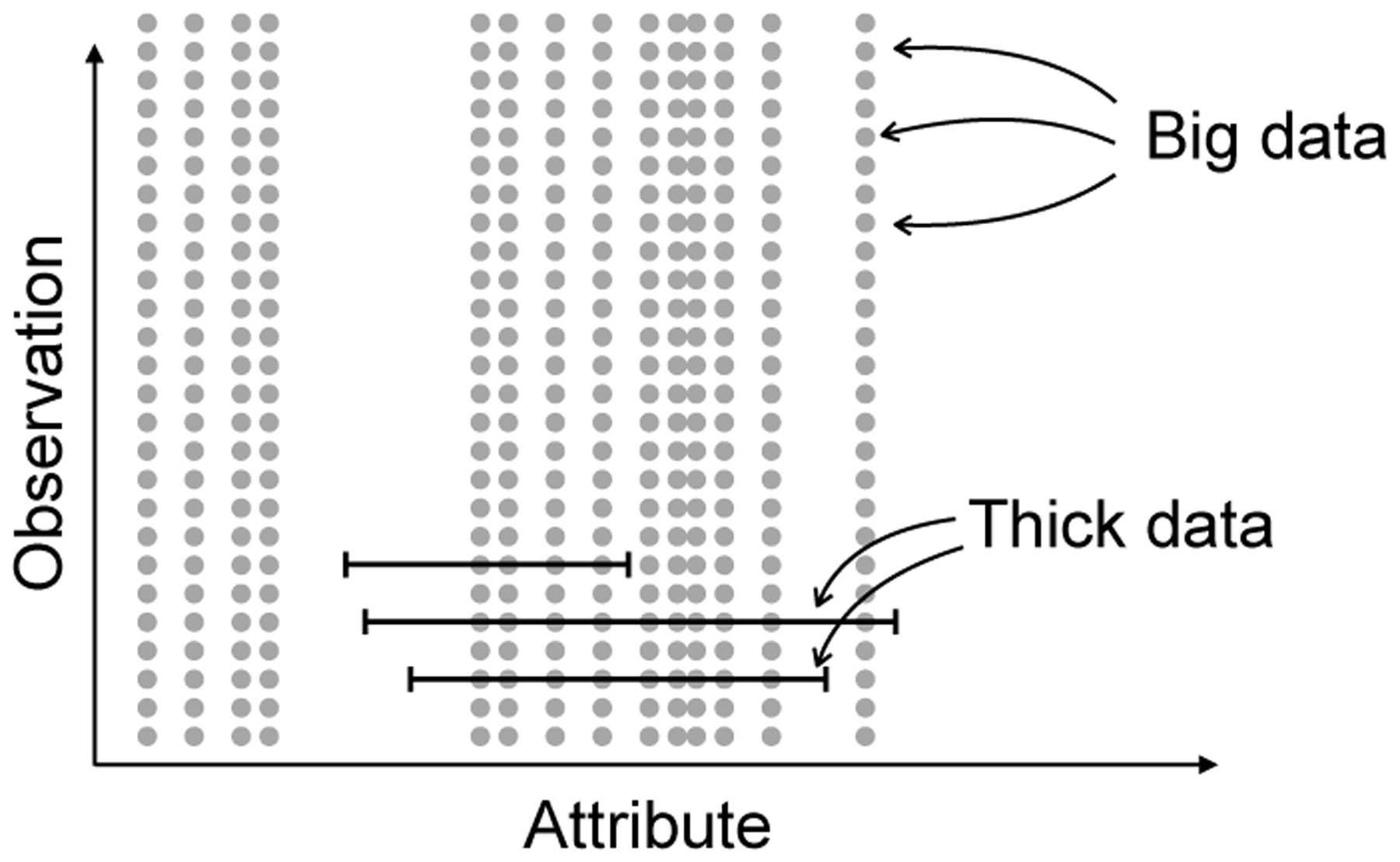
Distinction between big and thick data.

We illustrate the distinction between big and thick data in Fig. 2. Each row is an observation and the horizontal axis represents *X*, the set of attributes that determine outcomes. As shown in Fig. 2, big data used by algorithms and thick data used by humans differ in two fundamental ways. First, algorithms handle many more observations than people. Algorithms can read in thousands, millions, or even billions of cases of high dimensional data. A human’s experiences may be limited to a few dozen cases or perhaps a hundred cases. But humans also have potentially richer representations of those cases.

**Fig. 3:**
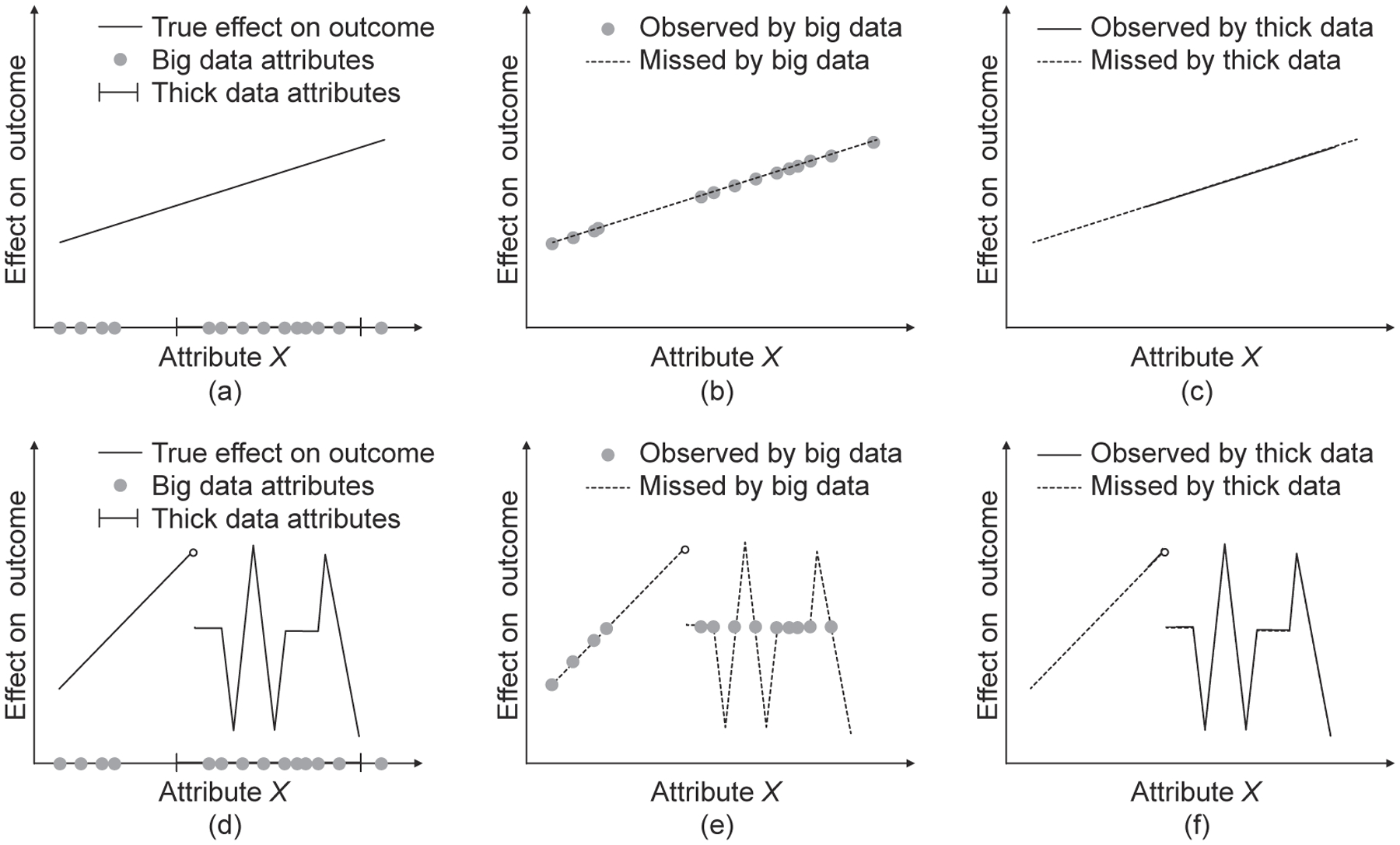
Trend of how big and thick data can fail to capture effects.

The relative contributions to predictive accuracy of big and thick data within a hybrid group depend on characteristics of the outcome function. That is to say, which data source is more effective, as well as how they might work in tandem to provide accurate predictions, depend on how attributes map to outcomes as shown through various cases in Fig. 3.

As can be seen from Fig. 3, both big data and thick data may fail to capture some of the attributes that impact outcomes. However, they tend to do so in different ways. Because big data are discrete, they can only cover a finite number of attributes. This has less of an effect on prediction when either only a finite number of attributes actually affect outcomes and the data capture most of the relevant attributes, or when the outcome function is relatively tame so that the impact of unobserved values can be accurately extrapolated from observed attributes. The top row of Fig. 3 depicts the latter case. Thick data that capture attributes in a continuous way, may omit ranges of the feature space.

The bottom row of Fig. 3 depicts a complex outcome function. In this case, big data accurately approximate the impact of some attributes, but miss other important factors by a wide margin. Predictions based on thick data are more likely to anticipate discontinuities or sudden jumps. This is because a person may possess a mental model that predicts a tipping point, where the value of a single attribute causes a major change in the outcome^[31]^.

As an example, consider an applicant for a mathematics PhD program. Big data might contain all of a student’s grades and standardized test scores, while thick data might be based on recommendation letters and an interview. A comment made in a letter “in recent weeks, she has made exceptional progress on a major unsolved problem in mathematics” might cause a different reaction to a human evaluator than to an algorithm which scores the letter on a scale from one to ten.

At the moment, thick data approaches may be better at capturing discontinuities —factors that have a large impact on outcomes that are not well approximated by other nearby attributes —while big data are better at including a large set and wider range of characteristics. These differences provide a hint that big data and thick data working together will produce more accurate collective predictions. Thick data can catch and draw attention to constellations of factors that night slip through the cracks between separated big data variables. Even though big data cast a wider net, that net contains holes. Ethnographers have long made similar arguments. A deep engagement involving the full repertoire of human senses, walking in another person’s shoes, produces a richer understanding.

Another strength of big data relative to thick data becomes clear when we consider how algorithms and humans make use of information contained in data. Both algorithms and humans use data on past outcomes to fit a predictive model for future outcomes. Error in these models can be decomposed into three components: *bias*, *variance*, and *irreducible error*.

Irreducible error is variability inherent in the outcomes that cannot be eliminated by any model, human or machine. Bias is error resulting from a difference between the functional form of the prediction and the true relationship between attributes and outcomes. For example, if the true outcome function, *F*, is quadratic and an algorithm allows for only linear functions, the algorithm’s predictions will be biased. Increasing the space of possible functions that an algorithm can fit reduces bias as one of those functions may be closer to *F*. This increase in model complexity comes at a cost; as the model becomes more complex, parameter estimates become more sensitive to the specifics of past observations inducing the third source of error, variance. When trained on more and larger datasets, algorithmic predictions exhibit low variance. Algorithmic predictors, like random forests and neural nets, possess almost limitless flexibility, so the amount of variance and how to balance it against bias become a choice variable rather than a constraint.

Human predictors suffer from the same trifecta of errors, but their relative contributions differ. The flexibility that humans possess in our mental models can reduce bias, but this flexibility along with the small number of cases we observe make overfitting a concern. As we construct more elaborate models in our head, we are less able to apply them to unfamiliar settings, resulting in potential bias. Human predictions also have substantial noise — they depend on mood, time of day, and even the weather – which may be an even larger source of error than bias^[32]^.

We can now apply this framework to see the impact of increasingly large datasets and more powerful algorithms. Clearly, these changes increase the accuracy of algorithms, but more important to our study of hybrid groups, as big data include more cases and attributes, correlations change. If, for example, there were no overlap in the dimensions considered by humans and algorithms, then predictions of algorithms and humans might be negatively correlated. In fact, in the special case of binary classifications, interpreted signals with non overlapping dimensions imply negative correlation^[23]^.

**Fig. 4:**
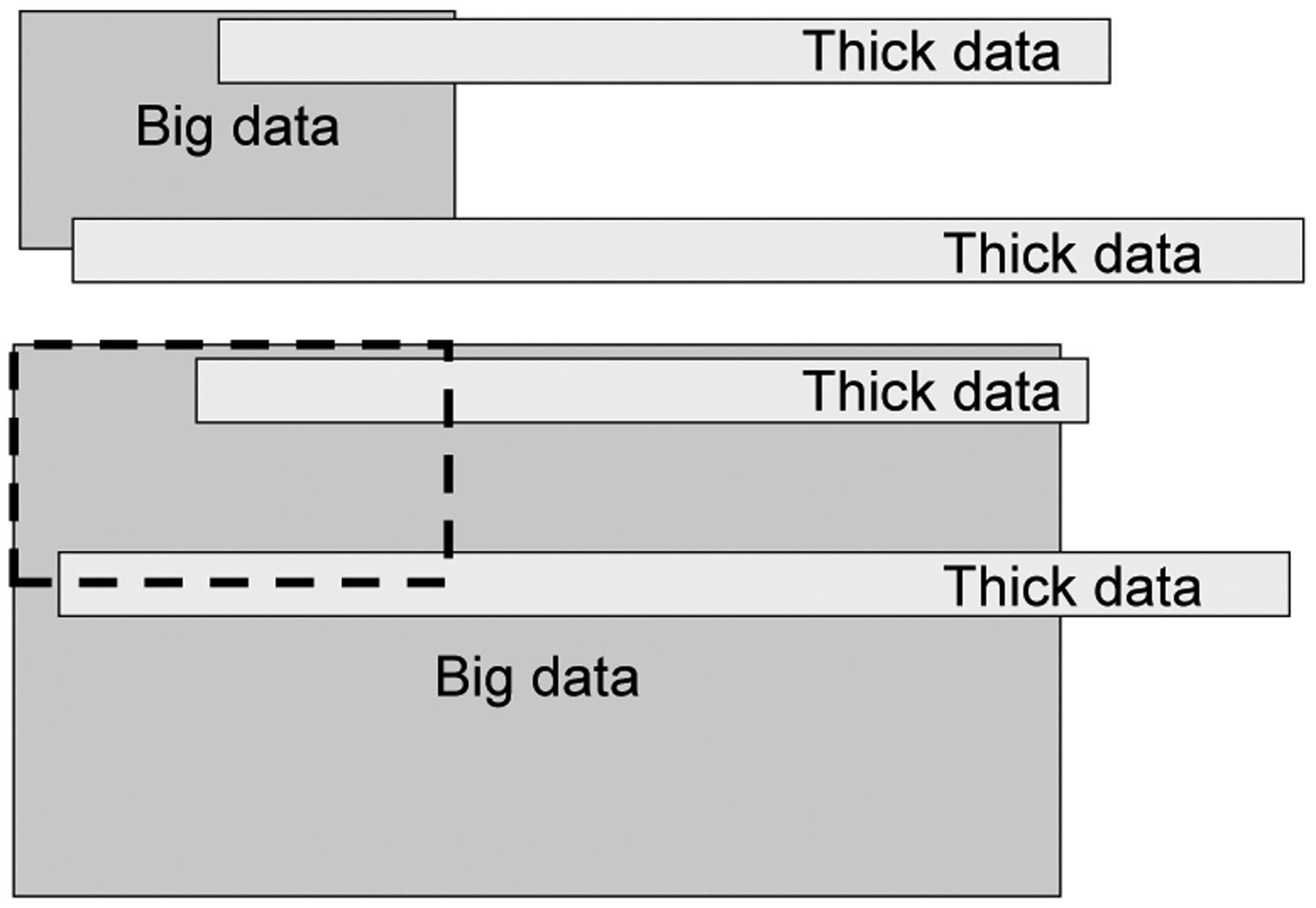
Increased overlap causing the accuracy-correlation effect.

As algorithms consider higher dimensional data, it likely overlaps more with the thick data used by humans as shown in Fig. 4. As the overlap increases, the correlation of the classifications should also become more positive. We refer to this as the accuracy-correlation effect (ACE).

### ACE:

As datasets become bigger, containing more cases and having more granularity, algorithmic predictions become more accurate and, if the overlap with thick data increases, it is more correlated with human predictions.

As we show formally in the next section, the accuracy-correlation effect greatly diminishes both how much humans contribute to accuracy and how much weight should be given to human predictions within a hybrid group.

### 3.1 Predictable and atypical cases

Many predictive tasks include cases in which context differs only marginally from the cases covered by big data and, in many cases, thick data. An example of a marginal change would be a shift in tax policy or environmental standards. In these cases, algorithms should outperform humans. However, cases can also be qualitatively different. Qualitative change would include natural disasters, financial meltdowns, new laws – for example, a requirement that all cars be electric by a certain date – and innovations.

Qualitative changes can, but need not, make past data irrelevant. We can distinguish between cases that are *predictable* given data and those in which existing data are not informative as predictors, i.e. *atypical* cases. We will assume that humans make more accurate predictions than algorithms in atypical cases because we can reason from analogy, imagine alternative futures, and understand causal mechanisms.

**Fig. 5:**
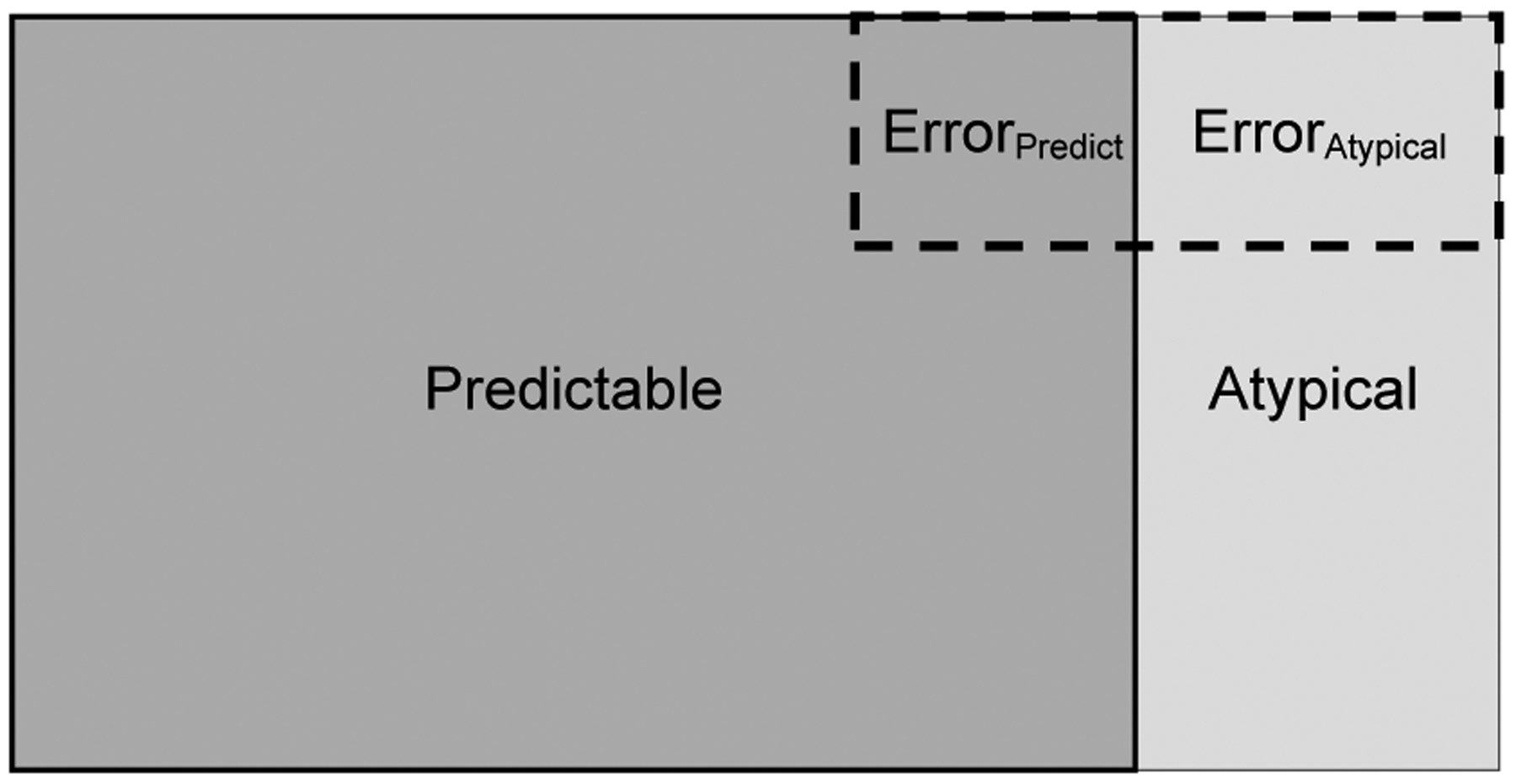
Predictable and atypical cases given big data (and classification errors).

The existence of atypical cases introduces a meta-predictive task, namely, determining whether an instance is predictable from data or not. Figure 5 partitions the set of all cases into two regions: *predictable* and *atypical*. Each region contains a subregion. Within the set of atypical cases, there exists a set of cases thought to be predictable (Error_Atypical_), and within the set of predictable cases, there exists a set of cases thought to be atypical (Error_Predict_).

### 3.2 Human + algorithm predictors

We analyze the potential value of hybrid predictions involving humans and algorithms using a *generated signal* framework. Our analysis relies on two well-known mathematical identities. The first, the *diversity prediction theorem* states that the crowd’s squared error equals the average individual squared error minus the diversity (variance) of the predictions. Suppose that the true value of an unknown quantity equals *V* and that there exists a collection of predictions denoted by *s*_*i*_ for *i* equals 1 to *N*. Let $\bar{s}$ equal the mean of these *N* predictions.

#### Diversity Prediction Theorem

(crowd error equals average error minus diversity):

$${\left(\bar{s}-V\right)}^{2}=\frac{1}{N}\overset{N}{\underset{i=1}{\sum }}{\left(s_{i}-V\right)}^{2}-\frac{1}{N}\overset{N}{\underset{i=1}{\sum }}{\left(s_{i}-\bar{s}\right)}^{2}.$$

We now apply this theorem to the special case of predictions of a single numerical value by a human (*h*) and by an algorithm (*c*). In what follows, we assume that the algorithm is more accurate than the human and, without loss of generality, that the human predicts a larger value (*h* > *c*). (This second assumption makes it so that we do not have to consider multiple cases.) Then the equation can be rearranged to the following.

#### Half the Distance Rule:

Given an outcome value *V*, the average of a human, *h*, and an algorithmic prediction, *c*, will be more accurate than the algorithm, if the squared error of the human minus the squared error of the algorithm is less than half the distance between the human’s and algorithm’s predictions,

$${\left(h-V\right)}^{2}-{\left(c-V\right)}^{2}<\frac{{\left(h-c\right)}^{2}}{2}.$$

The previous rule can be restated in terms of relative accuracy, where accuracy is defined as the inverse of the squared error. A squared error of ten corresponds to an accuracy of one-tenth. Define *Θ* > 1 to be accuracy of the algorithm relative to that of the human. If *Θ* = 3, this means that the humans squared error equals three times the squared error of the algorithm. We can then restate the *Half the Distance Rule* as follows.

#### Three Times Better Rule:

If the algorithm and human are equally like to err in the same or opposite directions, then the algorithm alone will be more accurate than an average of the human and the algorithm if and only if the algorithm is at least three times more accurate.

We first assume that the human and the algorithm are equally likely to err in the same direction or in the opposite direction. The expected squared error for the simple average of the human and the algorithm then equals follows (*α* below denotes the sqaure root of *θ*):

$$\frac{1}{2}\left(\alpha^{2}+1\right){\left(c-V\right)}^{2}-\frac{1}{8}\left[{\left(\alpha +1\right)}^{2}+{\left(\alpha -1\right)}^{2}\right]{\left(c-V\right)}^{2}=\frac{\alpha^{2}+1}{4}{\left(c-V\right)}^{2}.$$

This expression is less than the algorithm’s squared error, (*c*^2^ − *V*)^2^, if and only if *α*^2^ < 3.

Notice that the *Diversity Prediction Theorem* does not explicitly include bias and correlation, though they enter implicitly through the diversity term. To make the effects of bias and covariation explicit, we can invoke the *Bias-Variance Decomposition Theorem*. Keep in mind that when using the generated signal framework, we represent each prediction, whether by human or algorithm, as a random variable.

A random variable’s *bias* corresponds to the expected difference of the mean of that variable from the mean of the variable of interest. If on average, someone predicting electoral outcomes overstated the democratic party vote by 3%, then that person would have a bias of 0.03. For each pair of predictors, we can compute their *covariance*: the expected value of the product of each prediction from its mean. Two predictors with positive covariance generally err in the same direction and will be less accurate than two predictors with negative covariance.

The *Bias-Variance Decomposition Theorem* states that the expected error of an average of random variables (representing predictions) equals their average bias plus their average variance plus their average pairwise covariance. As above, let $\bar{s}$ denote the average of the *N* predictions, *s*_1_, and *s*_*N*_, and *V* denote the true value. Assume the average bias equals $\bar{\text{Bias}}(s)$, the average variance equals $\bar{\text{Var}}(s)$, and the average pairwise covariance equals $\bar{\text{Cov}}(s)$

#### Bias-Variance Decomposition Theorem:

Expected error equals average bias plus average variance plus sample covariance,

$$E\left[{\left(\bar{s}-V\right)}^{2}\right]=\bar{\text{Bias}}{\left(s\right)}^{2}+\frac{1}{N}\bar{\text{Var}}(s)+\left(1-\frac{1}{N}\right)\bar{\text{Cov}}(s).$$

In the case consisting of one human *h* and algorithmic predictor *c*, the equation can be written as

$$E\left[{\left(\frac{h+c}{2}-V\right)}^{2}\right]=\frac{{\left(\text{Bias}(h)+\text{Bias}(c)\right)}^{2}}{4}+\frac{\text{Var}(h)+\text{Var}(c)}{4}+\frac{\text{Cov}(h,c)}{2}.$$

The previous expression can be difficult to interpret. To build intuition, assume neither the human nor the algorithm has any bias. The degree to which the human can be less accurate but still contribute positively to accuracy in combination with the algorithm depends on the correlation between the predictions.

Notice that if the predictions become more negatively correlated, an even less accurate human still results in a hybrid pair that exceeds the accuracy of the algorithm alone. From the previous equation, we have

$$2\text{Bias}(h)\cdot \text{Bias}(c)+\text{Bias}{\left(h\right)}^{2}+\text{Var}(h)+2\text{Cov}(h,c)<3\text{Bias}{\left(c\right)}^{2}+3\text{Var}(c).$$

It follows that a simple average of the human and the algorithm will be more accurate if and only if *Var*(*h*) + 2*Cov*(*h*,*c*) < 3*Var*(*c*). Given this insight, we can then derinve analog of the *Three Times Better Rule* to include covariance.

#### Two to Six Rule:

If the algorithm and human’s covariance lies between minus one-quarter and one-quarter of the human variance, then the algorithm alone cannot be more accurate than an average of the human and the algorithm if the algorithm is only twice as accurate as the human, and the algorithm alone must be more accurate than the simple average if the algorithm is more than six times as accurate.

Recall from our analysis using interpreted signals that the more diverse the data that humans and algorithms use and the more different their models, the more likely they make negatively correlated predictions. If we take the degree of correlation as a proxy for the difference between humans and algorithms, that is, if we assume that thick data human predictions differ markedly from big data algorithmic predictions, then a hybrid prediction could outperform the algorithm even if the human were four or five times less accurate.

Moreover, if we relax the assumption of equal weighting and allow less weight on the less accurate human predictor, then the weighted average of the human and the algorithm will always be more accurate than the algorithm alone. However, as we place less weight on the human, we reduce the contribution of the human to collective accuracy. Even though thick data always add predictive accuracy under weighted averaging, it may add very little.

To see how little the human might add, assume that the human and the algorithm make independent predictions. In this case, the optimal weighting of the two predictions corresponds to their relative accuracies, that is, the optimal weightings on the algorithm and the human equal $\frac{\Theta }{1+\Theta }$ and $\frac{1}{1+\Theta }$, respectively. With these weightings, the expected squared error of the hybrid equals

$$E\left[{\left(\frac{\Theta c+h}{1+\Theta }-V\right)}^{2}\right]=\frac{\Theta \text{Var}(c)}{\left(1+\Theta \right)}.$$

which equals $\frac{\Theta }{\Theta +1}$ times the expected error of the algorithm.

Thus, given independent predictions, the expected squared error from a weighted combination of a human and an algorithm will always be less than the expected squared error of the algorithm alone. However, as is clear from the expression, the contribution of the human decreases in the relative accuracy of the algorithm. And, as discussed at length earlier, larger datasets and more powerful algorithms surely mean that *Θ*, the relative accuracy of the algorithm increases. And, yet, if we take the calculation above seriously, people remain relevant. Even if the computer algorithm was ten times as accurate as the human, the human would increase accuracy by nine percent, a non trivial amount.

The *accuracy-correlation effect* suggests that the independence assumption may be problematic. The weights to place on the human and the algorithm should therefore be adjusted to take into account covariance. The optimal weighting on the algorithm and the human, (*w*_*c*_, *w*_*h*_), are given by the following expression^[33]^:

$$\left(\frac{\Theta \text{Var}(c)-\text{Cov}(h,c)}{\left(1+\Theta )\text{Var}(c)-2\text{Cov}(h,c\right)},\frac{\text{Var}(c)-\text{Cov}(h,c)}{\left(1+\Theta )\text{Var}(c)-2\text{Cov}(h,c)\right)}\right).$$

It follows that the expected squared error given the optimal weighting is given by the following expression:

$$\frac{\Theta \text{Var}(c)\cdot \text{Var}(c)-\text{Cov}{\left(h,c\right)}^{2}}{\left(1+\Theta )\text{Var}(c)-2\text{Cov}(h,c\right)}.$$

Taking into account positive correlation places even less weight on the human. Of course, if correlation was negative, the human would get relatively more weight.

The fact that the weight on the human decreases in both the accuracy of the algorithm and in the correlation between algorithm and human implies that both parts of the *accuracy-correlation effect* reduce the contribution of the human predictor. In addition, accuracy and correlation have an interactive effect, thus further reducing the weight on the human predictor. To show this, we first work through an example and then create a parametrized family to show the shape of the effect. Assume that *Var*(*h*) = 6, *Var*(*c*) = 3(*Θ* = 2), and *Cov*(*h*, *c*) = −1. The optimal weights on the algorithm and the human are $\frac{7}{11}$ and $\frac{4}{11}$. The expected squared error of an optimally weighted hybrid group equals $\frac{17}{11}$, a reduction of just under 50%. Next, assume that the correlation switches from negative one to positive three-fourths. The optimal weights on the algorithm and the human become $\frac{7}{10}$ and $\frac{3}{10}$, and the hybrid’s squared error grows to $\frac{93}{40}$.

Weight on Human, **Hybrid Accuracy Improvement**:

**Table:** 

**Low Algorithm Accuracy**	
*Θ* = 2	
*Cov*(*h*, *c*) = −1,	(36%, **48**%);
*Cov*(*h*, *c*) = 0.75,	(30%, **23**%).

**Table:** 

**High Algorithm Accuracy**	
*Θ* = 6	
*Cov*(*h*, *c*) = −1,	(22%, **44**%);
*Cov*(*h*, *c*) = 0.75,	(5%, **1**%).

Now, assume that better data make the algorithm three times as accurate, so that *Var*(*c*) = 1. Assume that the correlation remains at negative one. The optimal weights become $\frac{7}{9}$ and $\frac{2}{9}$ with an expected squared error of $\frac{5}{9}$.

Finally, consistent with the *accuracy-correlation effect*, assume that when the algorithm becomes three times as accurate that the covariance switches from negative one to positive three-fourths: *Var*(*h*) = 6, *Var*(*c*) = 1, and *Cov*(*h*, *c*) = 0.75. This implies that *Θ* = 6 and the optimal weights on the algorithm and human equal $\frac{21}{22}$ and $\frac{1}{22}$.

In this last scenario, the weight attached to the human falls below 5%. The expected squared error of the weighted average of the algorithm and the human equals $\frac{87}{88}$, a negligible improvement over the algorithm alone. To see the magnitude of the interaction. If the effects were independent, the improvement on accuracy would be $\frac{1}{7}$, or approximately 14%. Instead, due to the positive correlation, adding the human only increases accuracy by 1%.

**Fig. 6:**
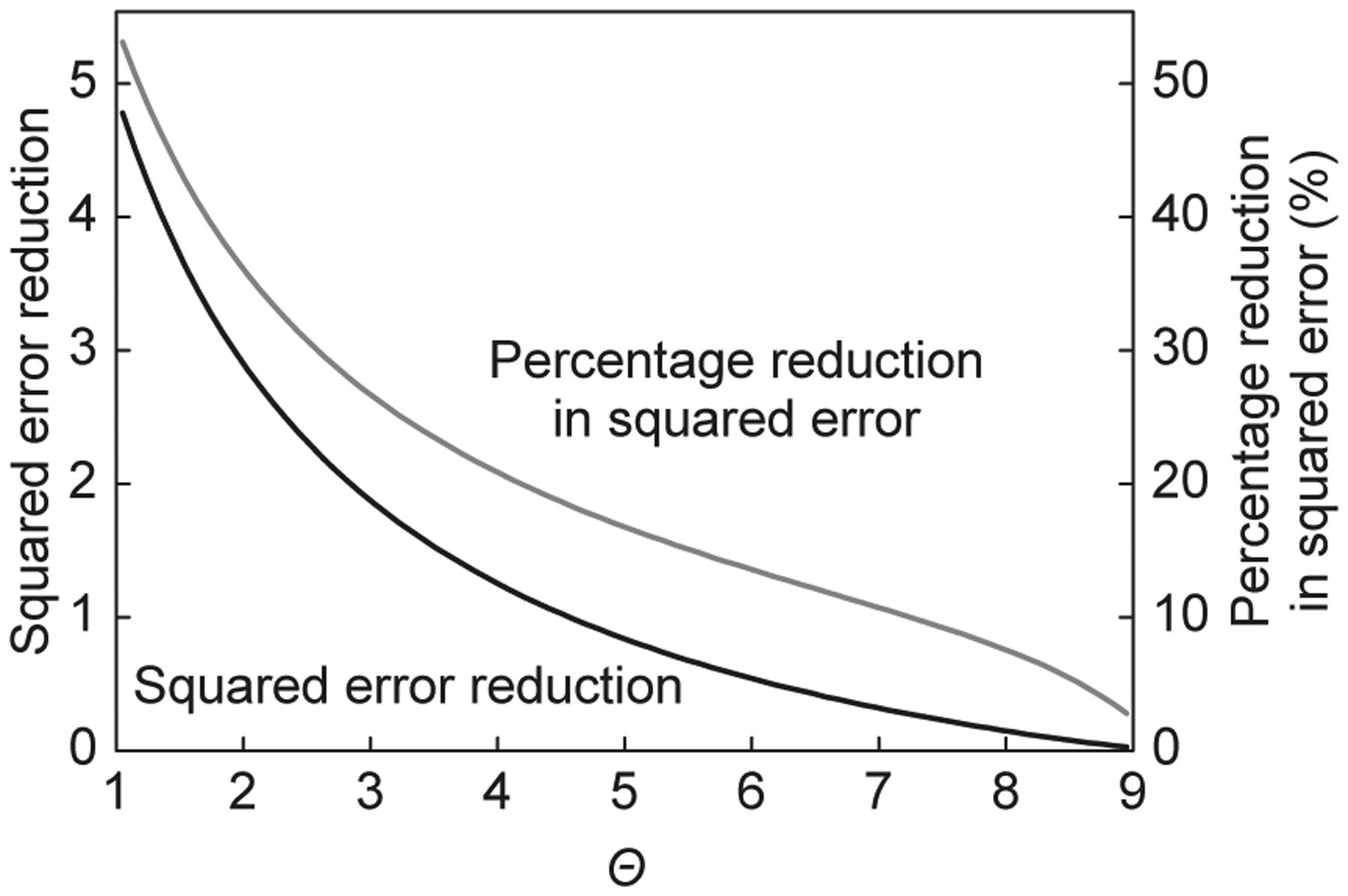
Contribution of human: *Var*(*h*)= 9, *Θ* ranges from 1 to 9.

To capture how the *accuracy-correlation effect* diminishes the contribution of human predictors more generally, we create a parametric family of algorithmic predictors. We first set *Var*(*h*) = 9, and let *Θ*, the relative accuracy advantage of the algorithm, lie in the interval^[1, 9]^. To embed the *accuracy-correlation effect*, we assume that the covariance takes the following form: $\text{Cov}(h,c)=\frac{\Theta -5}{8}$. We can then plot the reduction in squared error from adding the human and creating an optimal hybrid group. Recall that as *Θ* increases from 1 to 9, the squared error of the algorithm reduces from 9 to 1. We should therefore expect the absolute reduction in squared error to decrease. As shown in Fig. 6, the percentage reduction falls markedly as *Θ* increases because of the *accuracy-correlation effect*.

### 3.3 Predictability from data and atypical events

The cases we have considered so far assume that the big data are relevant to the predictive context. As discussed earlier, past data may not be of much use in predicting an outcome for a large change to a system such as an external shock or an attribute taking on an extreme value. Past data may also be of limited use when predicting the implications of a novel decision or strategic move. In these instances, humans may be more accurate than algorithms given the human capacities to draw analogies and to imagine alternative futures.

To make the choice to use a human rather than an algorithm requires being able to classify a prediction as *atypical* as opposed to *predictable from data*. The amount by which humans can improve accuracy on atypical events depends on how often atypical events must occur, how accurately they can be recognized, and on the relative accuracy of humans in those cases.

Ironically, identifying the set of atypical cases is itself a predictive task. The more transparent the algorithm, the better the human can predict that the algorithm is making a large mistake. Thus, as algorithmic performance improves, making algorithmic predictions more interpretable becomes more important^[34]^.

As already noted, deciding to abandon the algorithm involves a prediction that the case is atypical. And, whether abandoning is statistically supported depends on the accuracy of that prediction in a way that can be made precise. We restrict attention here to what we see as the most relevant case, that is in which the algorithm is more accurate than the human in the predictable region, and the human is more accurate than the algorithm in the atypical region.

Recall from Fig. 5, we need to consider four possibilities: (1) instances thought to be predictable from big data that are predictable, (2) instances thought to be predictable from big data that are atypical (Error_Atypical_), (3) instances thought to be atypical which are atypical, and (4) instances thought to be atypical which are predictable given big data (Error_Predict_).

If we assume the *accuracy-correlation effect*, the human predictor receives little weight in the hybrid prediction. Thus, the accuracy of hybrid prediction will be approximately the same as the algorithm’s alone. Therefore, in the predictable region minus Error_Predict_, hybrid prediction will be, to first approximation, the algorithm’s prediction. The same is true for instances thought to be predictable that turn out to be atypical (region Error_Atypical_). Whether or not to abandon the algorithm therefore hinges on the relative accuracies of the human and the algorithm in regions Error_Predict_ and the atypical region minus Error_Atypical_, the instances classified as predictable but that are actually atypical.

In the region Error_Predict_ outcomes are predictable given big data, and the human has a squared error equal to *Θ* times that of the algorithm. In that region, the human makes the prediction, which is a mistake. In the atypical region minus Error_Atypical_, the human also makes the prediction alone. In these cases, that is the correct decision. Whether abandoning the algorithm improves accuracy depends on the size of those regions relative to the sizes of predictive errors as stated below.

#### The Ratio Rule:

If the ratio of correctly identified atypical cases to incorrectly identified atypical cases exceeds the ratio of the product of algorithmic advantage, *Θ*, and algorithmic error on predictable cases to the product of human advantage on atypical cases *Ψ*, and human error on atypical cases, then letting humans predict cases identified as atypical reduces expected squared error iff the following holds,

$$\frac{\text{Prob}\left(\text{ Atypical }-{\text{ Error}}_{\text{Atypical }}\right)}{\text{Prob}\left({\text{Error}}_{\text{Predict }}\right)}>\frac{(\Theta -1)\text{Var}\left(c_{P}\right)}{(\Psi -1)\text{Var}\left(h_{A}\right)}.$$

If, for example, humans are ten times as accurate as the algorithm on atypical cases *Ψ* = 10, and algorithms are four times as accurate as humans on predictable cases, *Θ* = 4. If human error on atypical cases, *Var*(*h*_*A*_) equals two times *Var*(*c*_*P*_), then human need only be one-sixth as likely to correctly classify as an atypical case as it is to incorrectly classify a predictable case.

## 4 Discussion and Conclusion

We have presented an analysis of how humans and algorithms can jointly make better predictions than either alone in many instances. By taking a theoretical approach guided by empirical evidence, at the moment, hybrid groups should perform better than algorithms alone. So far as humans rely on thicker data than computers, they add more accuracy, and if there exists atypical events *and* even a crude ability to recognize them, humans could improve accuracy by reducing mistakes. This has the added advantage of building faith in hybrid predictors, and, counter-intuitively, in algorithms. By not allowing algorithms to make big mistakes, faith in algorithms increases.

Our formal analysis has considered a rather small crowd – one human and one algorithm. If we allow groups of people with exposure to different types of thick data and diverse algorithms (random forests and neural networks) based on different caches of data, both what we call the human and the algorithm would improve. Our entire analysis of a single human and a single algorithm could then be reinterpreted as representing a crowd of humans and an ensemble of algorithms with some caveats. With a group of humans, we might be able to further divide the atypical cases. Subject matter experts or specialists could be more accurate for specific atypical cases.

The question of whether humans will continue to be of value when making contributions merits deeper consideration. Continual technological advancements make datasets larger and larger and algorithms more sophisticated enabling algorithms to make increasingly accurate forecasts and classifications. Assuming there are only modest changes and improvements to how people predict, one could infer that the contributions of humans measured quantitatively would wane, with the caveat that we would still add value in atypical cases.

That line of thinking implies the necessity of greater interpretability of algorithmic predictions so that we can better predict the atypical. That will surely be the case. As implied by our formalism, if enhancing interpretability reduces misclassification of predictable and atypical instances, then it will yield significant increases in accuracy. It also follows that hybrid groups may be implemented more as an “or” than an “and,” by which we mean we may use AI in some cases and humans in others.

We believe these conclusions suffer from a failure to take into account human and AI’s adaptive capacities. First, recall that humans and AI differ in the information we can process, how we represent it, and how we derive predictions. Thus, we should not think of humans and AI as doing the same thing when making predictions. Second, humans learned to make predictions without AI as a potential partner. We develop protocols for how to predict with an eye toward improving individual accuracy through noise and bias reduction.

Those skills will remain useful, but they surely differ from the skills that would best complement AI. Through adaptation, humans can complement algorithms regardless of how wide and deep big data become. We can do so by learning to gather types of thick data and types of models that are accurate where the algorithms fail. We can think of how humans predict as the outcome of a cooperative game. Algorithm developers build the largest possible dataset and an ensemble of predictive models. Humans then explore and contemplate missing attributes or seek to identify conditions in which AI may fail. In the future, humans may well specialize on atypical cases – a form of adaptation to the algorithm. Overall, so long as humans can continue to identify different attributes, that is, continue to construct thicker data, or better understand atypical cases, they will continue to increase accuracy.

The cooperative game framing also reveals that algorithm designers should take into account the capacities of human predictive collaborators. The optimal algorithms to be part of a hybrid group might differ from algorithms design to make predictions without the aid of humans. Higher order algorithms may well guide humans as to what thick data they might gather by pointing to regions of low predictive confidence or limited interpretability. Rather than a competition between humans and computers, the future of hybrid predictors will be a complex search for symbiosis. The particulars cannot be known, but we can almost certainly predict that the roles and contributions of the participants will both adapt to ever growing data and greater computational power.

## Footnotes

§ Skidmore was an all women’s college until 1971.

## Appendix

### Proof of Half the Distance Rule:

Write the Diversity Prediction Theorem for *n* = 2 using *h* and *c* as the two predictions. This gives the following expression:

$${\left(\frac{h+c}{2}-V\right)}^{2}=\frac{1}{2}\left[{\left(h-V\right)}^{2}+{\left(c-V\right)}^{2}\right]-\frac{1}{4}{\left(h-c\right)}^{2}.$$

This can be rewritten as ${\left(h-V\right)}^{2}-\frac{1}{2}{\left(h-c\right)}^{2}<{\left(c-V\right)}^{2}$ which gives the result.

### Proof of Three Times Better Rule:

The magnitude of (*h* − *c*) depends upon whether *h* and *c* err in the same direction or opposite directions from the true value, *V*. Let *α* denote the square root of *Θ*. If, for example, both *h* and *c* exceed *V*, then (*h* − *c*) = (*α* − 1)(*c* − *V*). If they err in opposite directions, *h* > *V* > *c*, then (*h* − *c*) = (*α* + 1)(*V* − *c*).

### Proof of Ratio Rule:

Let *Var*(*x*_*P*_) denote the expected squared error of a predictor of type *x* = *h*, *c* in the predictable region and *Var*(*x*_*A*_) denote the expected squared error in the atypical region. Given our assumptions, *Var*(*h*_*P*_) = *ΘVar*(*c*_*P*_) and *Var*(*c*_*A*_) = *ΨVar*(*h*_*A*_). To simplify notation, let *E*_*P*_ and *E*_*A*_ denote the regions Error_Predict_ and Error_Atypical_. For large values of *Θ*, it follows that the hybrid–deference rule will have a lower expected squared error than an algorithm alone provided that Prob(*E*_*P*_)*Var*(*c*_*P*_) + Prob(Atypical − *E*_*A*_)*Var*(*c*_*A*_) exceeds Prob(*E*_*P*_)*Var*(*h*_*P*_) + Prob(Atypical−*E*_*A*_)*Var*(*h*_*A*_). This can be simplified as

$$\text{Prob}\left(\text{Atypical }-E_{A}\right)(\Psi -1)\text{Var}\left(h_{A}\right)>\text{Prob}\left(E_{P}\right)(\Theta -1)\text{Var}\left(c_{P}\right).$$

### Proof of Two to Six Rule:

$\text{Cov}(h,c)=\frac{-1}{4}\text{Var}(h)$ and $\text{Cov}(h,c)=\frac{1}{4}\text{Var}(h)$. Plugging these into the equation *Var*(*h*) + 2*Cov*(*h*, *c*) < 3*Var*(*c*) gives the result.
